# Tubercular Diseases of the Bones and Joints

**Published:** 1891-01-17

**Authors:** 


### SURGERY.

### TUBERCULAR DISEASES OF THE BONES AND JOINTS.

The nature of the processes which occur in so-called strumous affections of the joints and the contiguous portions of bone have been recently investigated by König, Baumgarten, Barker, Watson Cheyne, and others. There is an accumulation of evidence to show that these diseases are due essentially to the presence of the bacillus tuberculosis, although these germs may not always be found In his important lectures at the Royal College of Surgeons, Mr. Watson Cheyne says, "I attach no importance to the demonstration of tubercle bacilli as a means of diagnosing these bone and joint diseases;" but he considers that there are other characteristics which are more constant and essentially tubercular. He defines a tubercle histologically as "a microscopic nodule, generally round or oval in shape, composed of a central portion made up of epithelioid cells, and sometimes giant cells, surrounded by a layer consisting of cells of inflammatory origin, or of more completely formed fibrous tissue." This fibrous ring may or may not be the remains of a wall of a vessel, in which case the epithelioid cells, which are so characteristic, would probably be derived from the lining endothelium. This view of the origin of these masses is borne out by investigations on tubercular disease of the testis, and König lays stress on the fact that the disease in the articular ends of bones often leads to a conical mass of caseation or necrosis, suggestive of embolism, the small artery supplying the affected area being blocked by the formation of tubercles within its walls. The exact nature of the giant cells is not yet clear, and recent pathological work has not solved the question, some observers strongly advocating the view that the mass is one multinucleated cell; others (as Mr. Cheyne) considering their formation to be effected by either hypertrophy or coalescence of the characteristic epithelioid cells. The idea that in these affections the synovial membrane or the bone is the primary seat of the disease and not the articular cartilage is confirmed by modern investigation; but where cartilage merges into connective tissue, as in the inter-articular fibro-cartilages, there is evidence that the process may commence here, especially in the vertebral column. If the beginning is in the bone, the condition may be (1) miliary tuberculosis; (2) caseous deposits; (3) tubercular deposits with sclerosis; (4) superficial caries; (5) "dry" caries; (6) diffuse condensation; (7) diffuse softening; (8) tubercular periostitis and osteomyelitis. If in the synovial membrane (1) diffuse thickening; (2) limited nodules; (3) acute miliary tuberculosis; (4) König's "tubercular dropsy and empyæma of joints." "Strumous dactylitis" is an example of tubercular osteomyelitis, and Potts' disease is usually periostitis of the vertebræ. Mr. Arthur Barker reported at the Royal Medico-Chirurgical Society (October 28th) seven excisions of the hip in advanced cases which were treated by flushing with sterilised hot water. In six of these primary union occurred, and the disease appeared to be eradicated. "The evacuation was made so thoroughly that it was safe to close up the wound on the spot without drainage." As it is almost impossible to get rid of every particle of disease in such cases, the unusually good results obtained by Mr. Barker seem to indicate that the tubercular process is by no means spread over the whole or even the greater part of the diseased tissue; in fact that it is not usually diffuse but more or less localized. M. Chipault has recorded some remarkable results obtained by him in tubercular disease of the vertebral column treated by trephining and removal of the obviously affected bone tissue. Of thirty-five cases so treated there were twenty who were improved or definitely cured. The objects in this method of treatment are (1) to relieve the cord from the pressure of the inflammatory products, displaced bone, &c., and (2) to remove the tubercular centre, and so effect a local cure and prevent general inoculation of other parts. Besides these noticeable results in operative procedure very marked results have been obtained in Germany, and to some extent in England by the injection of Koch's fluid. Dr. Theodore Williams says, "The changes in the children in Professor Bergmann's and Dr. Levy's clinics impressed me greatly, the little fellows who had been cripples for months using their hip and knee joints quite freely." The inoculations appear to have produced marked improvement in some very severe cases, but probably operative interference will be required after this method has been practised in order to remove the sloughing and separated or encapsuled tubercular tissue.

